# Musculoskeletal pain and surgical ergonomics among orthopedic residents in Saudi Arabia: a cross-sectional study

**DOI:** 10.7717/peerj.21634

**Published:** 2026-08-24

**Authors:** Motaz Alaqeel, Abdulrahman Alaseem, Waleed Albishi, Zyad A. Aldosari, Mohammed N. Alhuqbani, Omar A. Aldosari, Badr F. Alshehri, Bandar I. Aljammaz, Ibrahim S. Alshaygy

**Affiliations:** Department of Orthopedic Surgery, King Saud University, Riyadh, Saudi Arabia

**Keywords:** Knowledge, Attitude, Practice, Ergonomics, Orthopedics, Musculoskeletal pain

## Abstract

**Background and aim:**

Poor surgical ergonomics may be associated with an increased risk of musculoskeletal pain (MSP) and injuries among surgeons in the workplace. We aimed to assess the knowledge, attitudes, and practices regarding surgical ergonomics and to determine the prevalence of MSP among orthopedic residents in Saudi Arabia. Methods: This research employed a cross-sectional design using a modified, validated tool distributed among orthopedic surgery residents. We performed tests of association between knowledge, attitude, and practice of surgical ergonomics and demographic and clinical characteristics, as well as their impact on the prevalence of MSP.

**Results:**

Among 158 orthopedic residents, 77.8% were male, with most in their first or second year of training. MSP was reported by 75.9%, predominantly affecting the lower back, feet, and neck. Trauma surgery was the most discomfort-provoking subspecialty (69%). MSP influenced fellowship decisions, mobility, and frustration tolerance. Changing posture during procedures was the most frequently used coping strategy. Greater knowledge of surgical ergonomics was associated with significantly lower odds of reporting moderate-to-severe MSP (odds ratio (OR) = 0.324, 95% CI [0.132–0.794], *p* = 0.014). A positive attitude toward surgical ergonomics was similarly associated with significantly lower odds of experiencing moderate-to-severe MSP (OR = 0.304, 95% confidence interval (CI) [0.108–0.855], *p* = 0.024).

**Conclusion:**

MSP was widespread among orthopedic residents and influenced both performance and career choices. Procedure-related pain impaired mobility and operative efficiency, while most residents relied on personal coping strategies in the absence of structured ergonomic support. Greater ergonomic knowledge and more positive attitudes were associated with lower odds of moderate-to-severe MSP, whereas reported practices showed no clear association. Despite broad support for formal ergonomics training, institutional provision remains limited. These cross-sectional study findings reflect associations rather than causality, underscoring the need for longitudinal research to better understand how ergonomic education and practice influence MSP over time.

## Introduction

Surgical ergonomics refers to the study and design of work environments and tools that optimize the well-being and performance of surgeons and surgical staff (Alaqeel & Tanzer, 2020). It aims to create an environment that promotes efficient and safe surgical practices while reducing the risk of musculoskeletal injuries and pain among surgical staff (Alaqeel & Tanzer, 2020). Surgical ergonomics should be implemented to prevent musculoskeletal diseases at work, which can lead to disability and impact the productivity and longevity of surgeons’ careers (Yurteri-Kaplan & Park, 2023).

Orthopedic surgeons are exposed to various occupational hazards, including cautery smoke, toxic chemicals, infectious diseases, needle-stick injuries, noise hazards, radiation, kidney stones, cataracts, infertility, musculoskeletal pain (MSP) or a combination of these (Ryu et al., 2021; Vajapey, Li & Glassman, 2021). While MSP is prevalent across all surgical specialties, it is most pronounced among orthopedic doctors (Alaqeel & Tanzer, 2020; McQuivey et al., 2021; Lucasti et al., 2022). Nearly two-thirds of orthopedic surgeons in Saudi Arabia have experienced a musculoskeletal condition related to their occupation, and 27% to 31% require time off ranging from half a day to forced retirement (Alzahrani et al., 2016; AlQahtani, Alzahrani & Harvey, 2016). Caseload and years of experience have been linked to a higher incidence of MSP among orthopedic surgeons (Alzahrani et al., 2016; AlQahtani, Alzahrani & Harvey, 2016). Although orthopedic residents have neither the caseload nor years of experience of full-time orthopedic surgeons, they often serve as surgical assistants and must hold extremities in awkward body positions, incurring muscular strain from prolonged retraction that primarily affects the lower back and upper extremity—the two most commonly involved areas (Alzahrani et al., 2016; AlQahtani, Alzahrani & Harvey, 2016).

Given these notable occupational hazards, improving orthopedic surgeons’ and residents’ knowledge, attitudes, and practices regarding optimal ergonomics may help mitigate the risk of MSP and occupational injury through the implementation of targeted ergonomic interventions (Valtanen, Van Niekerk & Chu, 2025). Studies have also shown a positive association between higher MSP and lower work satisfaction, burnout, and callousness toward others, with a substantial impact on professional behavior (McQuivey et al., 2021). Educating orthopedic surgery residents on these ergonomic interventions and enhancing their knowledge, attitudes, and practices contributed to safer surgical environments, greater well-being, and longer practice longevity, as well as improved patient care and a reduced burden of occupational injury (McQuivey et al., 2021; Valtanen, Van Niekerk & Chu, 2025). While it is generally recognized that practicing orthopedic surgeons experience higher rates of MSP due to their occupation, little research exists on MSP among orthopedic surgery residents. Although MSK disorders have been reported among both pediatric and adult consultant surgeons, this study is the first to assess the prevalence of MSK disorders, as well as the knowledge, attitudes, and practices regarding ergonomics among orthopedic surgery residents (Alqahtani, Alzahrani & Tanzer, 2016; Alzahrani et al., 2016; AlQahtani, Alzahrani & Harvey, 2016). This study was therefore conducted to determine the knowledge, attitudes, and practices regarding surgical ergonomics and the prevalence of MSP among orthopedic residents in Saudi Arabia.

## Methods

We conducted a cross-sectional study involving orthopedic surgery residents registered with the Saudi Commission for Health Specialties (SCFHS) in Saudi Arabia between January 2022 and December 2023. With the cooperation of SCFHS, an online survey was distributed to all orthopedic residents (R1 to R5) across all regions of Saudi Arabia. Ergonomic guidelines from the Canadian Center for Occupational Health and Safety (CCOHS), the National Institute for Occupational Safety and Health (NIOSH), and the Centers for Disease Control and Prevention (CDC) served as the basis for the modified survey tool, which was validated prior to use (Alaqeel & Tanzer, 2020; U.S. Department of Labor, 2025; National Institute for Occupational Safety and Health (NIOSH), 2025). Two orthopedic surgery consultants reviewed the survey tool for face and content validity, ensuring that the items were clearly constructed and adequately reflected the intended domains. A pilot test was then conducted among 10 orthopedic residents, with the survey administered twice, two weeks apart, to assess stability. Principal component analysis (PCA) was performed to identify correlated or redundant items and to define the underlying dimensions of the instrument. The final survey demonstrated strong internal consistency, with a Cronbach’s alpha of 0.82.

The survey tool consisted of three primary sections: (1) demographic information, including the number of hours worked and cases seen and performed annually; (2) a section focused on symptoms experienced in different body regions; and (3) a section assessing residents’ knowledge, attitudes, and practices related to surgical ergonomics. A score exceeding 66.7% of the maximum possible score was considered indicative of adequate knowledge, a positive attitude, or adequate practice (Boonstra et al., 2016). MSP was measured using the 11-point numeric rating scale (NRS), ranging from 0 (no pain) to 10 (severe pain). The NRS is a unidimensional measure of pain intensity that can be applied to the body as a whole or to specific body regions (Boonstra et al., 2016). An NRS of 1–3 was considered mild pain, 4–6 moderate pain, and 7–10 severe pain (Boonstra et al., 2016). Significant pain was defined as moderate-to-severe pain on the NRS scale.

The online survey was distributed *via* SurveyMonkey. Orthopedic surgery residents were contacted *via* email and invited to participate through program directors who distributed the survey among their residents. Sample size was calculated using the formula *n* = *Z*^2^^−^*p* (1 - *p*)/*e^2^*, where *n* is the sample size, *p* is the estimated prevalence, and *e* is the margin of error. Using a 95% confidence level, a 10% target proportion of the target population, and a 5% margin of error, the required sample size was 139 orthopedic surgery residents.

This study was granted approval by the institutional review board (IRB) of King Saud University Medical City (KSUMC) in Riyadh, Saudi Arabia (No. E-22-7423). All participants provided informed written consent after being made aware of the study’s objectives and purpose and were assured that their privacy and the confidentiality of information would be safeguarded through the removal of identifying details from the questionnaire.

Descriptive analysis was performed, with results expressed as numbers and percentages for categorical variables and as mean and standard deviation (for normally distributed variables) or median and interquartile range (for skewed variables) for continuous variables. Associations between demographic characteristics, MSP, and surgical ergonomics was assessed using the chi-square test. Comparison of means and standard deviations between two groups were conducted using the independent samples *t*-test (for normally distributed variables) and the Mann–Whitney test (for skewed variables). Normality of distribution was assessed using the Kolmogorov–Smirnov test. Multivariate regression analysis was performed on variables associated with MSP (*i.e.,* knowledge, attitude, and practice). Multicollinearity diagnostics, including the variance inflation factor (VIF), were performed on the variables included in the regression models to identify highly correlated variables and address potential issues with unreliable regression coefficients. All regression outputs, odds ratios (ORs), confidence intervals (CIs), *p*-values, and reference categories were independently rechecked against the original SPSS output files to ensure reporting accuracy and consistency throughout the manuscript, tables, and figures. All analyses were performed using the Statistical Program for Social Sciences (SPSS) version 26.0 (IBM, Armonk, NY, USA). Statistical significance was defined as a two-sided *p*-value < 0.05.

## Results

### Demographics and clinical characteristics

A total of 158 orthopedic residents completed the survey: 123 (77.8%) males and 35 (22.2%) females. The mean age was 28.1 ± 3.3 years old (range 18 –53 years). The majority were first- or second-year residents (36, 22.8% and 44, 27.8%, respectively). Participants spent a median of 10 h per week in the operating room (OR; Interquartile Range (IQR): 6.0, 15.0). The median number of cases anticipated annually was 100 (IQR: 42.0, 150.0). Mean body mass index (BMI) was 26.3 ± 4.7 kg/m^2^. Table 1 presents the detailed demographic characteristics of all 158 participants.

**Table 1:** Demographic characteristics of 158 orthopedic residents in Saudi Arabia.

Characteristics	Mean ± SD
Age in years, mean ± SD (95% CI, range)	28.1 ± 3.2 (27.5-28.6, 18–53)
BMI in kg/m^2^, mean ± SD (95%CI, range)	26.3 ± 4.7 (25.6–27.0, 16.8–42.5)
Gender, n (%)	
Male	123 (77.8%)
Female	35 (22.2%)
Region of practice, n (%)	
Central	62 (39.2%)
Western	46 (29.1%)
Eastern	33 (20.9%)
Southern	14 (8.9%)
Northern	3 (1.9%)
Residency level, n (%)	
1st	36 (22.8%)
2nd	44 (27.8%)
3rd	29 (18.4%)
4th	23 (14.6%)
5th	26 (16.5%)
Median (IQR) duration spent in the OR per week, in hours	10.0 (6.0–15.0)
Median (IQR) number of cases anticipated to be performed a year	100.0 (42.0–150.0)

### Symptomatology and pain characteristics

**Figure 1:**
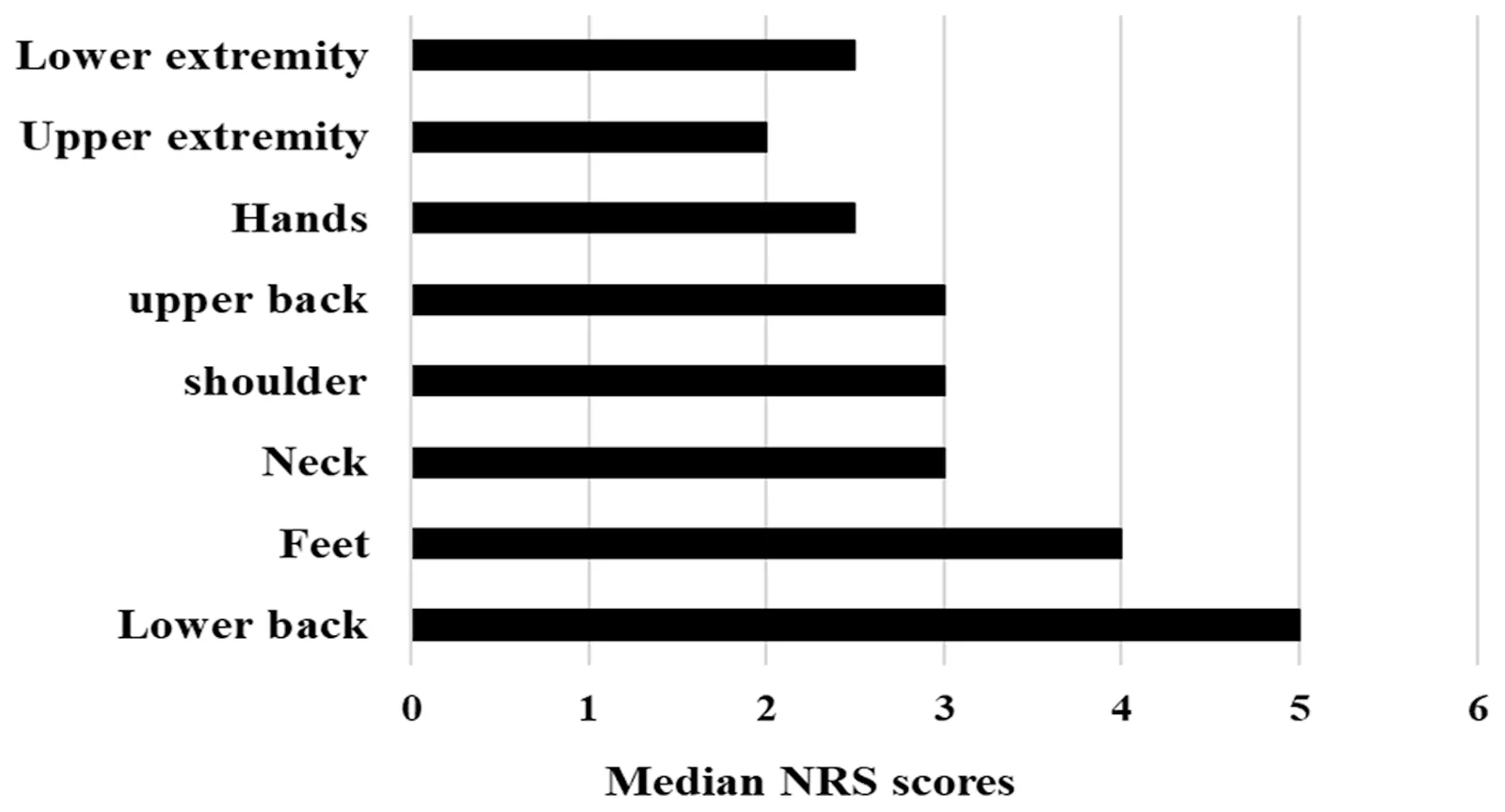
Anatomical locations where orthopedic residents experience MSP.

According to the NRS pain scale, 120 (75.9%) residents reported moderate-to-severe MSP. Median NRS scores were highest for the lower back (median: 5.0, IQR: 3.0–7.0), feet (median: 4.0, IQR: 2.0–6.0), neck (median: 3.0, IQR: 2.0–5.0), shoulder (median: 3.0, IQR: 0–5.0), and upper back (median: 3.0, IQR: 0–5.0; Fig. 1). There was no significant difference in the NRS scores by gender (*p* = 0.876), geographic location (*p* = 0.620), or year of training (*p* = 0.578) and no significant correlations with age (*r* =  − 0.022, *p* = 0.779) or BMI (*r* =  − 0.099, *p* = 0.216).

Residents who reported procedure-related MSP were more likely to allow it to influence important professional decisions. MSP appeared to weigh heavily on fellowship choice (OR 3.539, 95% CI [1.366–9.167], *p* = 0.009) and also interfered with the basic physical demands of the job. Residents with procedure-related MSP were more likely to experience difficulty with mobility during procedures (OR 5.226, 95% CI [1.181–23.117], *p* = 0.029) and to feel that their surgical speed was affected (OR 8.306, 95% CI [1.081–63.840], *p* = 0.042).

By contrast, several functional and behavioral domains showed no significant association with procedure-related MSP, including frustration intolerance (OR 6.107, 95% CI [0.785–47.507], *p* = 0.084), balance (OR 0.723, 95% CI [0.177–2.944], *p* = 0.650), concentration (OR 1.274, 95% CI [0.546–2.971], *p* = 0.575), operative approach (OR 3.000, 95% CI [0.368–24.479], *p* = 0.305), patience with others (OR 2.769, 95% CI [0.607–12.637], *p* = 0.188), degree of irritability (OR 1.476, 95% CI [0.589–3.698], *p* = 0.406), willingness to teach others (OR 0.471, 95% CI [0.144–1.539], *p* = 0.213), and stamina (OR 0.764, 95% CI [0.318–1.833], *p* = 0.547).

Most residents reported body discomfort within 1–6 h after a surgical procedure (*n* = 133, 84.2%); 109 residents (69.0%) experienced the most physical discomfort during trauma injury surgery, followed by spine surgery (*n* = 84, 53.2%) and arthroplasty procedures (*n* = 42, 26.6%). Ninety-four residents (59.5%) reported that their MSP began when they started residency training, and 84 (53.2%) reported that their condition worsened as a result of orthopedic residency. Forty-nine residents reported chronic MSP (31.0%), while 26 (16.5%) reported currently being in pain. Eleven (7.0%) reported a short-term disability, while four (2.5%) reported a long-term disability (Table 2).

**Table 2:** Characteristics of reported MSP and discomfort among 158 orthopedic residents.

Variables	n (%)
Onset of body discomfort attributable to surgery procedure	
Within an hour	14 (8.9%)
1–3 hour	55 (34.8%)
3–6 hours	78 (49.1%))
>6 h	11 (7.0%)
Area where they experienced the most physical discomfort	
Spine surgery	84 (53.2%)
Trauma injury surgery	109 (69.0%)
Arthroplasty	42 (26.6%)
Oncology	21 (13.3%)
Foot and ankle surgery	5 (3.2%)
Pediatrics	5 (3.2%)
Sports injuries	9 (5.7%)
Upper extremity surgery	4 (2.5%)
Experienced MSP when they started residency training	
Yes	94 (59.5%)
No	64 (40.5%)
Condition worsened by being an orthopedic resident	
Yes	84 (53.2%)
No	74 (46.8%)
Have chronic MSP	
Yes	49 (31.0%)
No	109 (69.0%)
Currently in pain	
Yes	26 (16.5%)
No	132 (83.5%)
Short-term disability	
Yes	11 (7.0%)
No	147 (93.0%)
Long-term disability	
Yes	4 (2.5%)
No	154 (97.5%)
Missed work	
Yes	2 (1.3%)
No	156 (98.7%)

### Knowledge, attitude, and practice of surgical ergonomics

A total of 133 residents (84.2%) demonstrated an adequate level of knowledge regarding surgical ergonomics. Knowledge scores did not differ significantly by age (*p* = 0.193), gender (*p* = 0.804), geographic location (*p* = 0.616), BMI (*p* = 0.323), or year of training (*p* = 0.756). Similarly, 141 residents (89.2%) demonstrated a positive attitude toward surgical ergonomics, with no significant variations across age (*p* = 0.536), gender (*p* = 0.636), geographic location (*p* = 0.935), BMI (*p* = 0.279), or year of training (*p* = 0.557). With respect to practice, 119 residents (75.3%) reported adequate adherence to surgical ergonomic principles. Practice scores likewise showed no significant differences by age (*p* = 0.536), gender (*p* = 0.466), geographic location (*p* = 0.698), BMI (*p* = 0.279), or year of training (*p* = 0.378). Additionally, no significant association was found between the prevalence of MSP and surgical ergonomic practice (*p* = 0.651).

### Surgical ergonomic interventions and behavior

**Figure 2:**
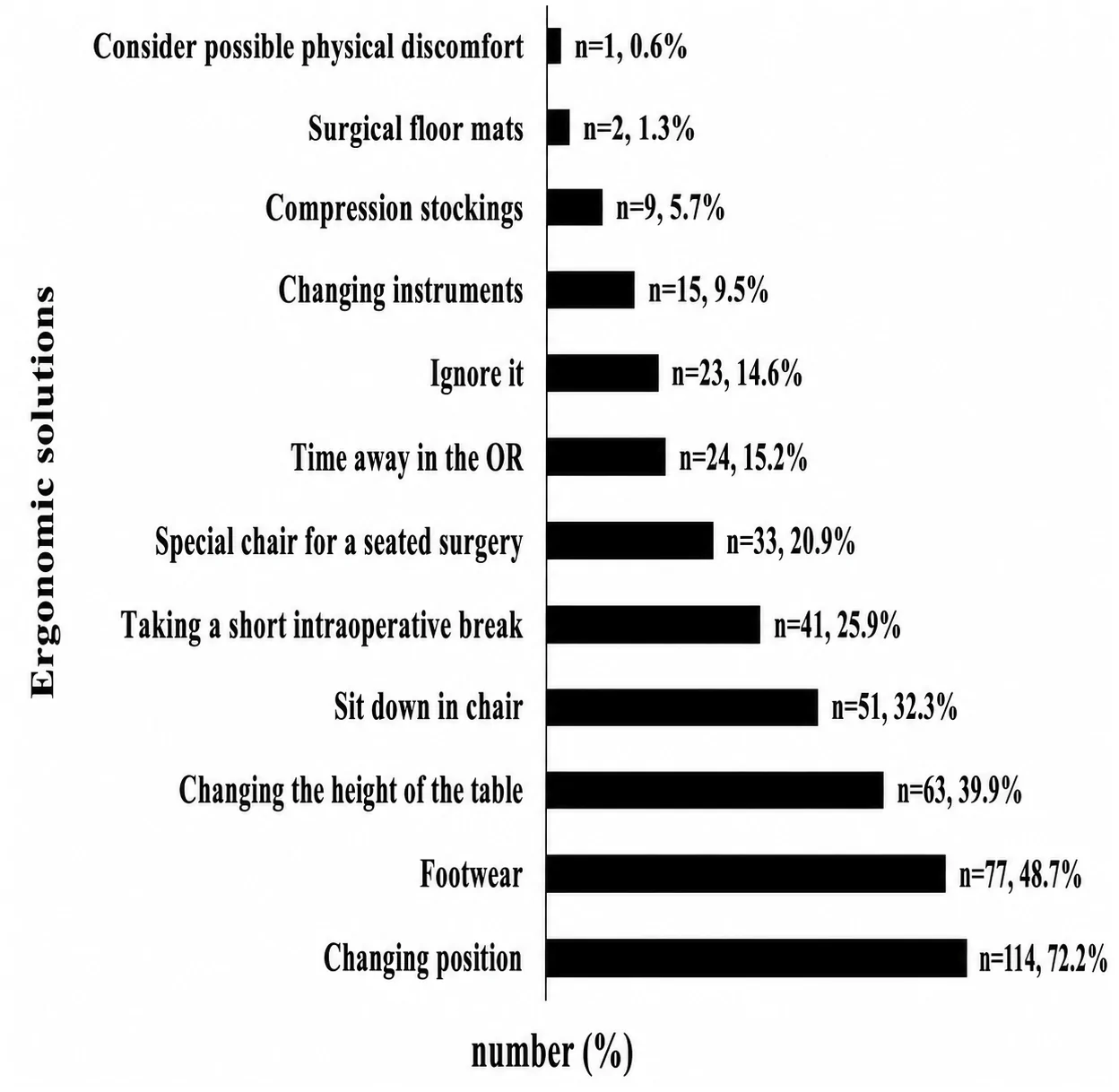
Ergonomic solutions to relieve pain among 158 orthopedic residents.

**Figure 3:**
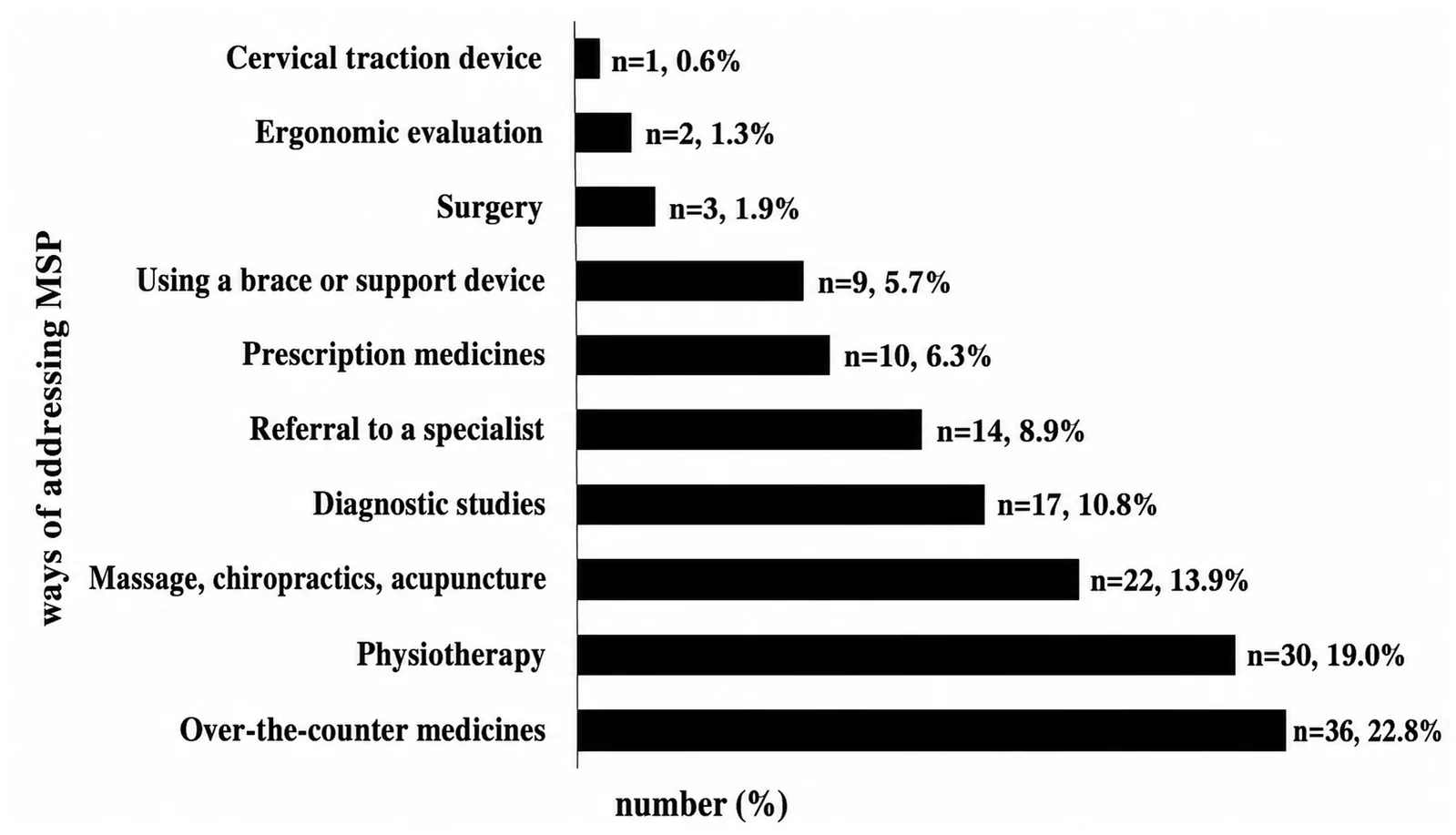
Various types of addressing MSP among 158 orthopedic residents.

Most orthopedic residents (*n* = 153, 95.8%) agreed that surgical ergonomics should be incorporated into residency training. They reported using various strategies to ease discomfort; the most common approach to managing MSP was changing position during surgery (*n* = 114, 72.2%), followed by wearing comfortable footwear (*n* = 77, 48.7%) and adjusting operating table height (*n* = 63, 39.9%; Fig. 2). Despite these individual efforts, ergonomic adaptations remained limited, and formal ergonomics training opportunities appeared to be scarce across orthopedic residency training programs in Saudi Arabia. Residents who reported MSP employed a range of management strategies. The most frequent approach was over-the-counter analgesics (*n* = 36, 22.8%), followed by physiotherapy (*n* = 30, 19.0%) and massage, chiropractic care, or acupuncture (*n* = 22, 13.9%; Fig. 3). Despite experiencing symptoms, six residents (3.8%) chose not to seek medical care and 14 (8.9%) informed the occupational health department of their condition. Two residents (1.3%) sought ergonomic evaluation.

### Impact of knowledge, attitude, and practice of surgical ergonomics on MSP

Greater knowledge of surgical ergonomics was associated with significantly lower odds of reporting moderate-to-severe MSP (OR 0.324, 95% CI [0.132–0.794], p = 0.014). After adjusting for age, gender, geographic region, year of training, case type, surgical duration, and ergonomics training, this association remained significant (OR 0.334, 95% CI [0.135–0.825], *p* = 0.017). Multicollinearity statistics showed VIF of 1.99 for age, 1.04 for gender, 1.07 for region, 2.19 for year of training, 1.21 for case type, 1.11 for duration, and 2.15 for ergonomics exposure.

A more positive attitude toward surgical ergonomics was similarly associated with significantly lower odds of experiencing moderate-to-severe MSP (OR 0.304, 95% CI [0.108–0.855], *p* = 0.024). This association remained significant after adjustment for age, gender, geographic region, year of training, case type, surgical duration and ergonomics-training exposure (OR 0.302, 95% CI [0.099–0.928], *p* = 0.037). Multicollinearity statistics for this model showed VIF of 1.96 for age, 1.04 for gender, 1.09 for region, 2.22 for year of training, 1.25 for case type, 1.09 for duration, and 2.09 for ergonomics exposure.

Adherence to surgical ergonomics practice was not significantly associated with the presence of MSP among orthopedic residents (OR 0.747, 95% CI [0.329–1.694], *p* = 0.485). The lack of association remained non-significant after adjustment for age, gender, geographic region, year of training, case type, surgical duration, and ergonomics-training exposure (OR 0.948, 95% CI [0.385–2.333], *p* = 0.948).

## Discussion

This study explored the spectrum of MSP experienced by orthopedic residents across Saudi Arabia and assessed their knowledge, attitudes, and practices regarding ergonomic strategies to address the issue. Most residents reported moderate-to-severe MSP, particularly involving the lower back, feet, and neck, and this pattern was consistent across age groups, genders, BMI categories, geographic regions, and year of trainings. Procedure-related MSP also appeared to influence several aspects of professional trajectory, including fellowship choices, mobility, frustration tolerance, operative technique, and surgical speed. Notably, residents with stronger knowledge and more positive attitudes toward surgical ergonomics were less likely to experience MSP.

Research on the practice and application of surgical ergonomics has expanded in response to the substantial burden of occupational injuries, hazards, and physical discomfort reported among orthopedic surgeons and other surgical specialists in the US, the UK, and several European countries (Valtanen, Van Niekerk & Chu, 2025; Morton & Stewart, 2022; Green et al., 2025; Gorce & Jacquier-Bret, 2024). The demanding physical and cognitive workload placed on surgeons, combined with challenges in maintaining work–life balance, has been consistently linked to high rates of MSP, with downstream effects on patient care, job satisfaction, and career longevity. These pressures may also shape early career decisions, including fellowship selection among residents, as reflected in the present findings (Ntani et al., 2025). Prior work has similarly noted that the high incidence of musculoskeletal injuries and MSP among surgeons can discourage medical students from pursuing surgical careers altogether (Sergesketter et al., 2019).

Consistent with the findings of McQuivey et al. (2021), this study documented a high burden of moderate-to-severe MSP among orthopedic residents, although the prevalence in the present cohort (75.9%) was slightly lower than their reported rate. Nonetheless, this estimate falls well within the broader prevalence range of 51.7%–97.0% described across multiple studies (McQuivey et al., 2021; Richard, Cho & Journeay, 2025). The demographic profile of the participants, particularly age and gender distribution, was comparable to that of the McQuivey cohort. However, the present sample included a larger proportion of first- and second-year residents, whereas more than half of their participants were in their first to third year of training.

Lower-level residents generally report fewer musculoskeletal symptoms than their senior counterparts, likely reflecting reduced cumulative exposure to physical stressors, greater physiological resilience, and the age-related degenerative changes that become more apparent in later years of training and practice (Al Mulhim et al., 2023). Although the observed prevalence of MSP, predominantly involving the lower back, feet, and neck, aligns with existing literature, this finding adds limited new insight given the methodological simplicity of the study. Consistent with prior reports, MSP was most frequently localized to the lower back and neck (Richard, Cho & Journeay, 2025). In contrast to earlier studies, however, this work did not identify associations between MSP prevalence and gender, younger age, or year of residency training (McQuivey et al., 2021; Grant, Vo & Tiong, 2020). Echoing these findings, several investigations have highlighted the broader consequences of MSP on surgeons’ physical and psychological well-being, including disruptions in sleep and overall quality of life (Scarcella et al., 2025).

Although most residents demonstrated strong knowledge, attitudes, and reported practices related to surgical ergonomics, the majority felt that formal ergonomics training should be incorporated into their residency programs. The findings show that 22.8% of residents relied on over-the-counter medications and physiotherapy to manage symptoms, whereas only 1.3% sought a formal ergonomic assessment. This gap likely reflects the limited opportunities for ergonomic adaptation in the operating room, as well as limited availability of structured ergonomics education within orthopedic residency training programs. These results underscore the need to translate strong knowledge and positive attitudes into consistent practice, closing the persistent knowledge-to-practice gap. Effective ergonomic practice is known to enhance surgical performance and reduce fatigue, pain, and injury (Karam & Soriano, 2025).

Punnett & Wegman (2004) revisited the longstanding debate over whether workplace physical exposures meaningfully contribute to musculoskeletal problems, particularly within the context of ergonomics. They examined the scientific evidence underpinning this discussion and noted that, although substantial data link repetitive tasks, forceful exertion, and suboptimal posture to MSP, skepticism persists, often attributed to the difficulty of distinguishing occupational from nonoccupational contributors. The authors emphasized the need for longitudinal research to better understand latency, natural history, prognosis, and potential selection bias and highlighted the absence of objective diagnostic tools for many symptoms, despite the value of subjective reporting in capturing the lived experience of pain (Punnett & Wegman, 2004).

In this context, the present study was designed primarily to document prevalence and assess knowledge and awareness, rather than to establish causation between workplace exposures and musculoskeletal disorders. We concur with Punnett & Wegman (2004) that several variables reported by our residents, such as the onset of MSP at the start of training or the worsening of symptoms during orthopedic residency, are inherently subjective and require longitudinal follow-up to determine whether they are truly work-related or influenced by nonoccupational factors. Moreover, although a statistically significant association was observed between greater ergonomic knowledge and attitudes and lower MSP, this association should not be interpreted as causal. Knowledge alone does not physiologically prevent pain; only changes in behavior or the working environment could plausibly alter risk.

The observed association between MSP and ergonomic knowledge should be interpreted cautiously. Surgeons experiencing work-related pain do not necessarily possess lower ergonomic knowledge; rather, the association may reflect broader structural and environmental barriers that limit the application of ergonomic principles in daily surgical practice. Multiple studies point to the limited presence of formal ergonomics education in medical training, and the physical demands of orthopedic practice routinely push surgeons into non-ergonomic positions, reducing both awareness and the ability to apply proper principles in real time (Buddle et al., 2023). Even when surgeons are generally aware of ergonomic concepts, this awareness does not reliably translate into consistent practice, particularly in operating rooms that lack ergonomically supportive equipment or in high-pressure surgical environments where ideal posture is difficult to maintain (Schlussel & Maykel, 2019). Linking pain exclusively to poor ergonomics without direct observational or biomechanical assessment remains speculative, as the relationship between discomfort and workplace setup is highly individualized and shaped by multiple interacting factors (Rashwan, Mohamed Sanad & Salah Eweida, 2024). Evidence from one meta-analysis underscores this complexity: although ergonomic interventions showed benefits for regions such as the lower back and neck, they did not significantly reduce pain in the thighs, arms, knees, shoulders, or elbows. Another study similarly reported that improvements in ergonomic awareness did not consistently translate into fewer sick-leave days or measurable gains in psychological well-being (Santos et al., 2025; Mahmud et al., 2011).

Feasible ergonomic strategies within residency programs include structured ergonomics education (*e.g.*, didactic teaching and hands-on workshops), along with training in posture and body mechanics, scheduled microbreaks, stretching routines, and practical adjustments to equipment and intraoperative workflow. These measures may involve simple modifications such as repositioning during procedures or using antifatigue mats that are directly relevant to the physical demands of orthopedic surgery (Schlussel & Maykel, 2019; Krishnanmoorthy et al., 2025; Winters et al., 2020; Beneka et al., 2024). Such interventions are designed to mitigate the substantial risk of work-related injury faced by surgical and interventional specialties, including orthopedics (Aaron et al., 2021). Beyond preventing acute strain, these approaches also target the cumulative toll of poor ergonomics, which can significantly affect residents’ physical well-being and ultimately threaten their long-term career trajectory (Perez et al., 2025). This concern was reflected in our findings, in which a notable proportion of residents reported contemplating a change in career path.

The findings of this study underscore the need for targeted ergonomic measures to help reduce MSP among surgeons. Incorporating ergonomics education into surgical training and embedding supportive workplace policies within hospitals would represent important steps toward preventing long-term functional impairment. In resource-limited settings, even modest adoption of ergonomic principles can meaningfully improve surgeon well-being, procedural efficiency, and ultimately the quality of patient care (Punnett & Wegman, 2004). To further strengthen surgical safety, institutions may also benefit from revisiting existing regulations and investing in ergonomics-focused research initiatives (Valtanen, Van Niekerk & Chu, 2025). Introducing coping strategies early in training can encourage healthier work habits and reduce ergonomic risks throughout a surgeon’s career (Noy, Shkedy & Vaisbuch, 2025). As procedure-related MSP has gained wider recognition, attention has increasingly shifted toward evaluating existing training approaches and identifying practical solutions. Only a limited number of surgical training programs currently incorporate formal or informal ergonomics instruction, and formalized ergonomics curricula remain particularly scarce in Saudi Arabia. This gap is often attributed to a lack of trained faculty and the absence of validated, evidence-based teaching models (Epstein et al., 2019). The persistence of MSP and surgeon discomfort, despite the availability of ergonomic recommendations and guidelines, underscores how entrenched the problem remains. Addressing MSP, especially among orthopedic trainees, is essential for reducing long-term morbidity, supporting career longevity, and sustaining interest in orthopedics as a profession. Establishing sound ergonomic habits early in training may help prevent the development of chronic MSP later in a surgeon’s career.

In this study, it is essential to distinguish between ergonomic exposure, ergonomic awareness, and pain prevalence, as each reflects a different aspect of the surgeon’s experience. Ergonomic exposure refers to the physical demands and postural stresses inherent to surgical work (*e.g.*, prolonged standing, repetitive motions, and suboptimal operating table height or positioning) (Liu et al., 2025). Ergonomic awareness, by contrast, reflects the surgeon’s understanding of ergonomic principles and the strategies available to reduce strain during procedures (McQuivey et al., 2021). Pain prevalence captures how many surgeons report musculoskeletal symptoms linked to their clinical duties. Clarifying these distinctions allows for a more accurate interpretation of how workplace conditions and individual awareness may shape the development of MSP among surgeons.

This study has several limitations. Although the survey was distributed to all orthopedic surgery residents across Saudi Arabia, only 158 responded, raising the possibility of type II error and limiting the power to detect meaningful differences or associations. Additionally, the sample may not fully reflect the broader orthopedic residency population, as self-selection bias is likely, with residents experiencing pain potentially more motivated to participate. We also recognize that some respondents had preexisting MSP, and not all symptoms described were necessarily attributable to orthopedic training alone; some may have reflected longstanding conditions exacerbated by operative demands. Future work should involve larger, more representative cohorts and explore ergonomic practices in greater depth to inform training curricula that emphasize proper intraoperative posture and increase awareness of occupational risks throughout surgical careers.

## Conclusion

Overall, MSP was widespread among orthopedic residents, often beginning early in training and affecting both day-to-day function and long-term career decisions. Residents with procedure-related pain were more likely to report difficulties with mobility and surgical pace, and many relied on self-directed strategies to cope. Despite this gap, most demonstrated strong knowledge and positive attitudes toward ergonomics, both of which were associated with lower odds of moderate-to-severe MSP. In contrast, reported ergonomic practices showed no meaningful relationship with pain. Although residents overwhelmingly supported integrating ergonomics into training, formal instruction and institutional support remain limited. As this study was cross-sectional, these findings reflect associations rather than causality, underscoring the need for longitudinal research to better understand how ergonomic education and practice influence MSP over time.

## Supplemental Information

10.7717/peerj.21634/supp-1 Supplemental Information 1 Questionnaire

## Additional Information and Declarations

### Data Availability

The following information was supplied regarding data availability: The data is available at Zenodo: Aldosari, Z. (2026). KAP SURGICAL ERGONOMICS [Data set]. Zenodo. https://doi.org/10.5281/zenodo.18405754
