# Supplementary material for: Musculoskeletal pain and surgical ergonomics among orthopedic residents in Saudi Arabia: a cross-sectional study
Source: PeerJ. 2026 Aug 24;14:e21634. doi: 10.7717/peerj.21634 (PMC13525743; doi:10.7717/peerj.21634)
Supplement: Supplementary file 1 [file peerj-14-21634-s001.pdf]

KAP of Surgical Ergonomics

Knowledge, Attitude, and Practices Towards Surgical Ergonomics Orthopedic Residents in Saudi Arabia

You are invited to this online survey for a study titled "Knowledge, Attitude, and Practices Towards Surgical Ergonomics Among Orthopedic Residents in Saudi Arabia"

This study aims to assess the knowledge, attitude, and practices of orthopedic residents toward surgical ergonomics.

All the responses will be confidential. Filling out this survey is completely voluntary, and will take approximately (5) minutes to complete.
If you have any questions feel free to send them to this e-mail address: knowledgeattitudepractice@gmail.com
This research is supervised by Dr.Motaz Alaqeel(1), Dr.Ibrahim Alshaygy(1)
(1) Assistant Professor at collage of medicine KSU, Consultant of Orthopedics KKUH

* 1. Please tick this check box:

- [ ] I am agreeing and willing to participate in this research study without conferring or referring to the literature , and I am aware that my answers will be used for research purposes.

* 2. Please tick this check box:

- [ ] I am agreeing and willing to participate in this research study without conferring or referring to the literature , and I am aware that my answers will be used for research purposes.

KAP of Surgical Ergonomics

Section #1: Demographics:

testing testing 123

* 3. Q1: How old are you? (Age)

____

* 4. Q2: Are you ?(Gender)

- ○ Male
- ○ Female

* 5. Q3: How tall are you? (Height in cm)

____

* 6. Q4: How much do you weigh? (Kg)

* 7. Q5: Are you (Dominant hand)
  - Right handed
  - Left handed

* 8. Q6: Where do you practice?
  - Central
  - Eastern
  - Northern
  - Southern
  - Western

KAP of Surgical Ergonomics

Section #2: Residential program:

* 9. Participants PGY year
  - PGY-1
  - PGY-2
  - PGY-3
  - PGY-4
  - PGY-5
  - PGY-0

* 10. Approximately (on average), how many hours do you spend in the operation room (OR) in a typical week?

* 11. How many cases do you anticipate to perform by the end of year?(annually)

* 12. Q5: How often do you wear lead?

- ◯ Every case
- ◯ Every day
- ◯ 2-3 days a week
- ◯ At least once a week
- ◯ At least once a month
- ◯ Other (please specify)

KAP of Surgical Ergonomics

Section #3: Surgical Ergonomics Knowledge

* 13. How much do you agree to these statements?

	Agree	to some extent	Disagree
I know what is meant by surgical ergonomic practice?	○	○	○
I know the benefits of optimizing surgical ergonomics practices?	○	○	○
I know the health hazards of my job without surgical ergonomics practices?	○	○	○
Ergonomics principles try to change the worker behavior and not to change the working environment.	○	○	○
Although surgical ergonomics are beneficial to improve worker health, it costs more money than what saves.	○	○	○
Keeping the shoulders relaxed could cause Procedural related Musculoskeletal Pain	○	○	○
Musculoskeletal Pain may be caused by the type of grip for long periods	○	○	○
Frequent use of mallets > 1.75 kg at work contributes to Procedural related Musculoskeletal Pain	○	○	○
Footwear plays an important role in preventing lower extremity complaints.	○	○	○
The surgeon should work while maintaining a neutral joint posture as much as possible.	○	○	○

KAP of Surgical Ergonomics

Section #4 Surgical Ergonomics attitude

* 14. How much do you agree with these statements

	Agree	To some extent	Disagree
Ergonomics education must be a part of the residency program	◯	◯	◯
I prefer to bend my head forward instead of adjusting the workspace for better viewing	◯	◯	◯
I would prefer to bend my back while working because it makes my work easier	◯	◯	◯
Forceful hand movements while working enables me get work done on time even if it could hurts	◯	◯	◯
Doing exercises like stretching, walking etc. are important to be more productive at work	◯	◯	◯
I should always adjust the table hight according to the task being done	◯	◯	◯
I am overall satisfied with my work	◯	◯	◯
I feel that the procedural related musculoskeletal pain experienced increase my callousness toward others	◯	◯	◯
I feel that the procedural related musculoskeletal pain experienced increase my burnout	◯	◯	◯
I feel that the procedural related musculoskeletal pain that I experience in the OR will influence my ability to do surgical procedures in the future.	◯	◯	◯
The procedural			

related musculoskeletal pain experienced during a specific rotation plays a notable role in deciding which fellowship I will pursue.	○	○	○
I feel that wearing lead contributes to increased pain while performing surgery	○	○	○

* 15. I worry about the effects of radiation on my (Mark all that apply.)

- ☐ Eyes
- ☐ Brain
- ☐ Thyroid
- ☐ Breast
- ☐ Reproductive organs
- ☐ Other (please specify)

* 16. Do you believe wearing lead contributes to increased pain while performing surgery?

- ○ Yes
- ○ No

17. Were you pregnant while performing orthopedic cases requiring fluoroscopy?(females participants part)

- ○ Yes
- ○ No

18. While pregnant did you? (females participants part)

- ○ Use ‘Double Lead’ during pregnancy
- ○ Use ‘Maternity Lead’ during pregnancy when performing fluoroscopy cases
- ○ Avoid cases requiring fluoroscopy during pregnancy

KAP of Surgical Ergonomics

Section #5 Surgical Ergonomics practice

* 19. Please answer the following statements :

	Yes	Sometimes	No
When you are in the OR, do you wear comfortable shoes?	◯	◯	◯
Do you find enough space to be in a comfortable posture?	◯	◯	◯
Are your hands or arms subjected to pressure from sharp edges on work surfaces/tools?	◯	◯	◯
Do all task requirements are visible from comfortable positions?	◯	◯	◯
Do you avoid twisting or bending your wrist?	◯	◯	◯
Do you stop every 60 minutes to get up to stretch and move for 1-2 minutes?	◯	◯	◯
While you are holding objects (e.g., Forceps), do you alternate between your fingers holding them? (ex. Thumb and index or index and middle finger)?	◯	◯	◯
Do you place the patient as close as possible to you on the operating room bed?	◯	◯	◯
Do you tilt the bed toward you to maximize the operative field visualization and minimize both the need to lean and bend over the patient.?	◯	◯	◯

* 20. Do you change the table height based on

- ◯ Your height
- ◯ The Primary surgeon's height
- ◯ The assistant surgeon's height
- ◯ I don’t change the table height

KAP of Surgical Ergonomics

Section #6: Ergonomic related pain/Procedural related Musculoskeletal Pain (MSP)

* 21. How much work-related physical discomfort or pain do you typically experience after a full day of performing procedures or operating from a scale of 10?

0 5 10

* 22. If you have had ANY work-related body discomfort in the past 30 days, check the body part with how much discomfort you have on a scale of 0 to 10, where 0 is “None” and 10 is “Worst imaginable”

	None 0	1	2	3	4	5	6	7	8	9	Worst imaginable 10
Neck	◯	◯	◯	◯	◯	◯	◯	◯	◯	◯	◯
Shoulder	◯	◯	◯	◯	◯	◯	◯	◯	◯	◯	◯
Upper back	◯	◯	◯	◯	◯	◯	◯	◯	◯	◯	◯
Lower Back	◯	◯	◯	◯	◯	◯	◯	◯	◯	◯	◯
Hands	◯	◯	◯	◯	◯	◯	◯	◯	◯	◯	◯
Feets	◯	◯	◯	◯	◯	◯	◯	◯	◯	◯	◯
Upper extremity(excluding hands)	◯	◯	◯	◯	◯	◯	◯	◯	◯	◯	◯
Lower extremity (excluding feet)	◯	◯	◯	◯	◯	◯	◯	◯	◯	◯	◯

* 23. If you have had ANY work-related body discomfort in the past 30 days, check the body part with how much discomfort you have on a scale of 0 to 10, where 0 is “None” and 10 is “Worst imaginable”

	None 0	1	2	3	4	5	6	7	8	9	Worst imaginable 10
Neck	◯	◯	◯	◯	◯	◯	◯	◯	◯	◯	◯
Shoulder	◯	◯	◯	◯	◯	◯	◯	◯	◯	◯	◯
Upper back	◯	◯	◯	◯	◯	◯	◯	◯	◯	◯	◯
Lower Back	◯	◯	◯	◯	◯	◯	◯	◯	◯	◯	◯
Hands	◯	◯	◯	◯	◯	◯	◯	◯	◯	◯	◯
Feets	◯	◯	◯	◯	◯	◯	◯	◯	◯	◯	◯
Upper extremity(excluding hands)	◯	◯	◯	◯	◯	◯	◯	◯	◯	◯	◯
Lower extremity (excluding feet)	◯	◯	◯	◯	◯	◯	◯	◯	◯	◯	◯

* 24. On average, how long after the start of an operation or procedure do you begin to experience body discomfort attributable to surgery?

◯ Within 1 hour

◯ 1-3 hours

◯ 3-6 hours

◯ >6 hours

* 25. Have you experienced symptoms of any musculoskeletal conditions/injury since starting residency, and if so, how have they been treated?

◯ No

◯ Yes, pursued no treatment

◯ Yes, pursued non-operative treatment

◯ Yes, pursued operative treatment

* 26. Do you contribute your condition to your work as an orthopedic resident?

◯ Likely work-related

◯ Likely not work-related

* 27. Was your condition worsened by your work as an orthopedic resident?

◯ Yes

◯ No

* 28. In your experience, which rotations are associated with the most physical discomfort in the OR?
(Mark all that apply.)

- [ ] Spine
- [ ] Trauma
- [ ] Arthroplasty
- [ ] Oncology
- [ ] Foot and ankle
- [ ] Pediatrics
- [ ] Sports injuries
- [ ] Upper extremity

* 29. Does the amount of physical discomfort you experience in the OR during a specific rotation play a role in deciding which fellowship you will pursue?

- ○ Yes
- ○ No

* 30. Does your pain or discomfort affect any of the following? (Mark all that apply.)

- [ ] Sleep
- [ ] Relation with others
- [ ] Consumption of over-the-counter- drugs
- [ ] Prescription drug intake
- [ ] Surgical posture
- [ ] Balance
- [ ] Concentration
- [ ] Mobility
- [ ] Operative approach
- [ ] Patience with others
- [ ] Degree of irritability
- [ ] Intolerance or Frustration
- [ ] Willingness to teach others
- [ ] Stamina
- [ ] Surgical speed
- [ ] Tremor
- [ ] None of the above

* 31. How have you attempted to minimize work-related physical discomfort? (Mark all that apply.)

- [ ] Changing Position
- [ ] Taking a short intraoperative break
- [ ] special chair for seated surgery
- [ ] Set up tool/Sit down chair
- [ ] changing the height of the table
- [ ] Changing instruments (example long vs short micro instruments)
- [ ] Time away from the OR.
- [ ] Consider possible physical discomfort or symptoms when choosing an operative approach for an individual patient.
- [ ] Footwear
- [ ] Compression stockings
- [ ] Surgical floor mats
- [ ] Ignore it

Other (please specify)

* 32. If you have had CHRONIC (more than 30 days) surgical/procedural-related pain (please mark all that apply).

- [ ] I do not have chronic pain
- [ ] Currently in pain
- [ ] Sought medical care
- [ ] Short-term disability
- [ ] Long-term disability
- [ ] Missed work
- [ ] Ergonomic evaluation

* 33. Please mark all types of medical help that you sought for these aches, pain, or discomfort. (Mark all that apply.)

- ☐ Referral to a specialist (neurology, orthopedics, physical medicine and rehabilitation, etc.)
- ☐ Diagnostic studies (X-ray, CT, MRI, etc.)
- ☐ Physiotherapy
- ☐ Prescription medicine
- ☐ Over-the-Counter Medication
- ☐ Surgery
- ☐ Using a brace or support device
- ☐ Cervical traction device
- ☐ Massage, chiropractic, acupuncture therapy
- ☐ I have not sought medical help for these aches, pains, or discomfort

Other (please specify)

* 34. Have you ever reported your condition(s) to occupational health or employee health resources at your place of work?

- ○ Yes
- ○ No
- ○ My employer does not have employee or occupational health resources
- ○ Not Applicable
